# Editorial: European partnership on the assessment of risks from chemicals (PARC): continued focus on new approach methodologies (NAMs) and data gap filling

**DOI:** 10.3389/ftox.2026.1903700

**Published:** 2026-07-16

**Authors:** Anna Beronius, Erwin Roggen, Terje Svingen

**Affiliations:** 1 Institute of Environmental Medicine, Karolinska Institutet, Stockholm, Sweden; 2 3Rs Management and Consulting ApS, Lyngby, Denmark; 3 National Food Institute, Technical University of Denmark, Lyngby, Denmark

**Keywords:** chemical testing, data gap filling, NAM, regulation, toxicology

Chemical risk assessment stands at a critical inflection point. Although numerous legislative systems are in place for assessing the potential of industrial chemicals to cause harm to human health and the environment, current approaches supporting regulatory decision-making remain insufficient to address the scale and complexity of modern chemical exposures. The continued introduction of new substances and evolving exposure scenarios challenge the traditional risk assessment paradigms, thus motivating a shift toward next-generation risk assessment (NGRA) that makes greater use of new approach methodologies (NAMs) in place of animal models. This transition is driven by scientific and regulatory needs, as well as broader societal and ethical commitments to reduce animal use for toxicity testing.

Despite progress, key challenges remain for NGRA (Fadeel et al., 2025). For instance, the integration of heterogeneous data into robust, regulatorily accepted frameworks is inherently complex. There are also persistent data gaps, alongside limited mechanistic understanding of adverse outcomes, which continues to constrain both hazard assessment and the uptake of NAMs. Addressing these limitations requires not only methodological innovation, but also targeted data generation to support regulatory decision-making and build confidence in emerging approaches.

These scientific priorities are now reinforced by evolving European policy. In June 2026, the European Commission adopted a roadmap to phase out animal testing for chemical safety assessments, outlining concrete actions to accelerate the development, validation, and uptake of non-animal approaches across regulatory domains (European Commission, 2026). This policy framework aims to position NAMs as central to future safety assessment strategies while maintaining a high level of protection for human health and the environment.

Within this context, the European Partnership on the Assessment of Risks from Chemicals (PARC; https://www.eu-parc.eu/), a 7-year initiative launched in 2022, provides a key framework to support the transition toward NGRA (Marx-Stoelting et al., 2023). As PARC is past its halfway point, the focus is shifting from prioritization toward implementation, yet maintaining core activities aimed at developing applicable NAMs. Work Package 5 (WP5) on hazard assessment plays a central role by advancing NAMs while also addressing data gaps for chemicals of regulatory concern; two closely interconnected objectives that underpin the transition toward more predictive and less animal-dependent risk assessment (De Castelbajac et al., 2023).

This Research Topic builds on earlier PARC collections and highlights ongoing WP5 activities across key toxicological domains (Ramhøj et al., 2024). In the following sections, we introduce the individual contributions and outline how they advance the implementation of NAMs and the closing of critical data gaps in chemical risk assessment.

The Research Topic comprises seven manuscripts. Four introduce new PARC projects, one presents a tiered testing strategy, one experimental study provides new data on endocrine-disrupting chemicals (EDCs), and one offers an overview of PARC prioritization for the latter stage of the programme. The latter (Arenas et al.) outlines how second-round priorities were refined, including consideration of the Key Areas of Regulatory Challenge defined by the European Chemicals Agency, ensuring alignment with evolving regulatory needs.

The first new project (Dirven et al.) describes PlasticLeach, which addresses data gaps for the large number of insufficiently characterized chemicals associated with plastics (Monclús et al., 2025). Using non-targeted analytical approaches followed by hazard testing, the project aims to identify and characterize toxic leachates and support both regulatory decision-making and the development of safer materials. The second project (Ardenkjær-Skinnerup et al.) focuses on advancing NAMs for developmental and reproductive toxicity (DART) testing with focus on endocrine disrupting effects, a particularly challenging area for replacing animal testing. Activities include predictive modelling, refinement of alternative organism models, and development of *in vitro* systems targeting key tissues such as testis, placenta, and brain.

The third project (Smith et al.) addresses developmental immunotoxicity (DIT), aiming to refine understanding of critical windows of immune system development and support NAM-based risk assessment. By leveraging the AOP framework (Edwards et al., 2016) and literature synthesis, the project will develop an integrated physiological map and support regulatory applications such as screening and read-across. The fourth project (Billat et al.) focuses on inhalation exposure, combining *in vitro* and *in silico* approaches to study uptake, internal exposure, and systemic availability. Using data-rich substances such as PFAS, the project integrates read-across and physiologically based kinetic modelling to improve predictive capacity.

The experimental study (Vandeputte et al.) identifies zebrafish neuromast development as a target of EDCs, showing that compounds affecting estrogenic and thyroid pathways impair embryonic development at environmentally relevant concentrations. The final perspective (Aiello et al.) presents a tiered hazard assessment strategy combining OECD test guidelines and NAMs to evaluate bisphenol A (BPA) alternatives across multiple endpoints. This work illustrates how coordinated NAM-based testing can support NGRA and help prevent regrettable substitutions.

Together, the contributions in this Research Topic exemplify the transition from conceptual development to practical implementation of NAM-based approaches within PARC. By combining methodological innovation with targeted data generation and regulatory alignment, these studies contribute to advancing a more integrated, predictive, and ethically grounded framework for chemical risk assessment.
